# A PKC-MARCKS-PI3K regulatory module links Ca^2+^ and PIP_3_ signals at the leading edge of polarized macrophages

**DOI:** 10.1371/journal.pone.0196678

**Published:** 2018-05-01

**Authors:** Brian P. Ziemba, Joseph J. Falke

**Affiliations:** Department of Chemistry and Biochemistry, and the Molecular Biophysics Program, University of Colorado at Boulder, Boulder, CO, United States of America; Indiana University School of Medicine, UNITED STATES

## Abstract

The leukocyte chemosensory pathway detects attractant gradients and directs cell migration to sites of inflammation, infection, tissue damage, and carcinogenesis. Previous studies have revealed that local Ca^2+^ and PIP_3_ signals at the leading edge of polarized leukocytes play central roles in positive feedback loop essential to cell polarization and chemotaxis. These prior studies showed that stimulation of the leading edge Ca^2+^ signal can strongly activate PI3K, thereby triggering a larger PIP_3_ signal, but did not elucidate the mechanistic link between Ca^2+^ and PIP_3_ signaling. A hypothesis explaining this link emerged, postulating that Ca^2+^-activated PKC displaces the MARCKS protein from plasma membrane PIP_2_, thereby releasing sequestered PIP_2_ to serve as the target and substrate lipid of PI3K in PIP_3_ production. *In vitro* single molecule studies of the reconstituted pathway on lipid bilayers demonstrated the feasibility of this PKC-MARCKS-PI3K regulatory module linking Ca^2+^ and PIP_3_ signals in the reconstituted system. The present study tests the model predictions in live macrophages by quantifying the effects of: (a) two pathway activators—PDGF and ATP that stimulate chemoreceptors and Ca^2+^ influx, respectively; and (b) three pathway inhibitors—wortmannin, EGTA, and Go6976 that inhibit PI3K, Ca^2+^ influx, and PKC, respectively; on (c) four leading edge activity sensors—AKT-PH-mRFP, CKAR, MARCKSp-mRFP, and leading edge area that report on PIP_3_ density, PKC activity, MARCKS membrane binding, and leading edge expansion/contraction, respectively. The results provide additional evidence that PKC and PI3K are both essential elements of the leading edge positive feedback loop, and strongly support the existence of a PKC-MARCKS-PI3K regulatory module linking the leading edge Ca^2+^ and PIP_3_ signals. As predicted, activators stimulate leading edge PKC activity, displacement of MARCKS from the leading edge membrane and increased leading edge PIP_3_ levels, while inhibitors trigger the opposite effects. Comparison of the findings for the ameboid chemotaxis of leukocytes with recently published findings for the mesenchymal chemotaxis of fibroblasts suggests that some features of the emerging leukocyte leading edge core pathway (PLC-DAG-Ca^2+^-PKC-MARCKS-PIP_2_-PI3K-PIP_3_) may well be shared by all chemotaxing eukaryotic cells, while other elements of the leukocyte pathway may be specialized features of these highly optimized, professional gradient-seeking cells. More broadly, the findings suggest a molecular mechanism for the strong links between phospho-MARCKS and many human cancers.

## Introduction

Leukocytes, including macrophages and neutrophils, possess a sophisticated chemosensory pathway that adeptly directs cellular migration up attractant gradients while seeking infections, tumors, or wounds (reviewed in [1–13]). Leukocytes are characterized by stable polarization even in the absence of an attractant gradient, with most components of the chemosensory pathway localized to the membrane at the leading edge of the cell. The leading edge chemosensory pathway directs random migration until an attractant gradient appears, then directs migration up the gradient. Both stable polarization and gradient sensing require a leading edge positive feedback loop long known to include members of the Ras superfamily of small G proteins, isoforms of the lipid kinase phosphoinositide-3-kinase (PI3K), the PI3K-produced signaling lipid phosphotidylinositol-(3,4,5)-triphosphate (PIP_3_), and regulators of actin polymerization [1–13]. More recently, it was discovered that Ca^2+^ and protein kinase Cα (PKCα) are also localized to the leading edge where they are essential components of the leukocyte positive feedback loop [14,15]. Similarly, in chemotaxing fibroblasts, the PKCα activators Ca^2+^ and diacylglycerol (DAG) are localized to the leading edge [16–19]. In short, extensive evidence now indicates that leading edge PKCα activity is essential to both the ameboid chemotaxis of leukocytes and the mesenchymal chemotaxis of fibroblasts [8,14–19].

Prior studies of polarized macrophages revealed that stimulation of the Ca^2+^ signal triggers dramatic amplification of leading edge PI3K activity and rapid accumulation of its product signaling lipid PIP_3_, as well as expansion of the leading edge region [14]. Moreover, inhibition of Ca^2+^ influx through plasma membrane channels blocks the leading edge PIP_3_ signal and collapses the leading edge [14]. These findings, plus the demonstration that leading edge Ca^2+^ is also a central player in neutrophil chemotaxis, have demonstrated a strong link between Ca^2+^ signaling, PIP_3_ signaling, and leading edge growth control in leukocyte ameboid chemotaxis [8,14,15,20]. Models have proposed that Ca^2+^-activated PKCα, together with the abundant signaling protein myristoylated alanine-rich C kinase substrate (MARCKS [21,22]) could provide the previously unknown regulatory link between the leading edge Ca^2+^ and PIP_3_ signals [8,14,20]. These models suggest that MARCKS binding downregulates PIP_3_ production via its ability to tightly bind and sequester multiple (up to four) molecules of plasma membrane phosphotidylinositol-(4,5)-diphosphate (PIP_2_), which serves as both the membrane target and substrate lipid of PI3K. Subsequently, PI3K would be activated when the sequestered PIP_2_ is released by PKCα phosphorylation of 3 specific sites on MARCKS [21,23], or by CaM binding to an overlapping target region on MARCKS [24,25], thereby increasing the local pool of accessible PIP_2_ for PI3K membrane binding and lipid kinase activity. Recent *in vitro* single molecule studies of the reconstituted pathway on supported lipid bilayers mimicking the plasma membrane surface demonstrated that MARCKS binding to PIP_2_ does indeed inhibit PI3K activity [20,26]. Moreover, Ca^2+^-activated PKCα or CaM displaces MARCKS from PIP_2_ thereby restoring PI3K activity and PIP_3_ production [20,26].

**Fig 1:**
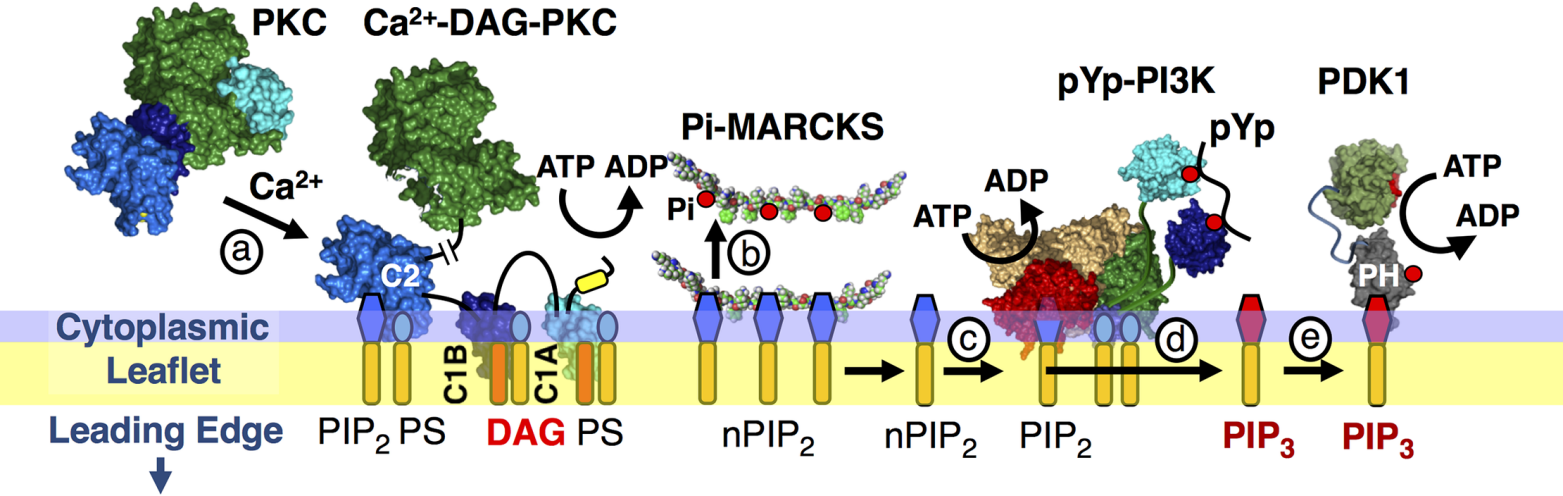
Schematic molecular model of hypothesized signaling events linking Ca^2+^ and PIP_3_ signaling at the leading edge of polarized leukocytes. The model [8,14,20] proposes that Ca^2+^ signals can stimulate PIP_3_ production by **(a)** driving Ca^2+^-PKC binding to membrane where it is activated by DAG (produced by Ca^2+^-PLC, not pictured). The resulting Ca^2+^-DAG-activated PKC **(b)** phosphorylates MARCKS and displaces it from sequestered PIP_2_ [20,26]. Following MARCKS displacement, the increased density of free PIP_2_ on the membrane surface provides additional target lipid binding sites that **(c)** recruit additional active PI3K molecules to the membrane surface, thereby **(d)** increasing net PI3K lipid kinase activity and PIP_3_ production. The increased PIP_3_ levels in turn **(e)** drive increased recruitment of PIP_3_- specific PH domain proteins, including PDK1, to the membrane surface.

Further studies in live leukocytes are needed to test the hypothesized leading edge Ca^2+^- PKCα-MARCKS- PIP_2_-PI3K-PIP_3_ signaling module (Fig 1). Previous studies of macrophages and neutrophils confirm a number of the model predictions [2,14,27–34]), but the linkage between Ca^2+^ signaling, PKCα activity, MARCKS localization to the leading edge plasma membrane, and PI3K-catalyzed production of leading edge PIP_3_ has not yet been directly tested in leukocytes. The present study employs live cell fluorescence imaging of macrophages to systematically examine the effects of two activators (attractant stimulus, Ca^2+^ influx) and three inhibitors (PKC inhibitor, PI3K inhibitor, and Ca^2+^ influx inhibitor) on four leading edge membrane activities (PIP_3_ production at the leading edge, MARCKS recruitment to the leading edge membrane, leading edge PKCα activity, and leading edge expansion/contraction). The results define a perturbation matrix of 20 effects on leading edge physiology. Overall, the findings provide strong evidence of the central importance of the Ca^2+^- PKCα-MARCKS- PIP_2_-PI3K-PIP_3_ signaling module in regulating leading edge expansion and contraction in polarized leukocytes. The findings, together with other evidence in the field, yield a more comprehensive model of positive feedback in ameboid cell migration.

## Results

### The predictions tested

The Ca^2+^-PKCα-MARCKS-PIP_2_-PI3K-PIP_3_ signaling module hypothesis predicts that activators of the leading edge positive feedback loop will stimulate PI3K and PIP_3_ production, and will also increase PKCα activity and phosphorylation of MARCKS [8,14,20]. Since MARCKS phosphorylation by PKCα drives dissociation from PIP_2_, the phospho-MARCKS population will release sequestered PIP_2_ and dissociate from membranes. At the other extreme, inhibitors of the positive feedback loop are predicted to downregulate PI3K and PIP_3_ production, and to decrease PKCα activity and MARCKS phosphorylation, thereby shifting the MARCKS population towards membrane association and PIP_2_ sequestration.

### The three sensors employed

Three sensors were employed to test these predictions by monitoring the effects of activators and inhibitors on (i) PI3K production of PIP_3_, (ii) PKC activity, and (iii) MARCKS binding to the membrane, all at the leading edge of polarized, actively ruffling macrophages. AKT-PH (pleckstrin homology) domain is a standard PI3K activity sensor [14,35,36] that specifically binds plasma membrane PIP_3_ even in the presence of PIP_2_. This domain was fused to the fluorescent protein mRFP to yield the PIP_3_ sensor AKT-PH-mRFP. Protein kinase C activity reporter (CKAR) is a soluble PKC activity sensor that possesses a specific target sequence phosphorylated by PKC and an intramolecular CFP-YFP FRET output that reports the phosphorylation state [37]. The MARCKSp-mRFP sensor possesses a peptide corresponding to the PIP_2_ binding region of MARCKS, and is known to bind to the plasma membrane surface until its target Ser residues are phosphorylated by PKC [20,23,28,34,38–41]. The control sensor MARCKSp-SA4-mRFP possesses a Ser to Ala mutation at each of the four PKC target sites, thereby allowing its membrane binding, but preventing its phosphorylation and membrane displacement [42]. Each sensor was expressed individually because leukocytes in general, and macrophages in particular, are front line immune cells that efficiently reject foreign DNA making transfection with multiple constructs difficult or impossible [43,44].

A significant advantage of sensor studies of signaling at the leukocyte leading edge is the straightforward identification of cells that both express the chosen sensor and retain a functional leading edge pathway. For each of the four sensors, confocal fluorescence microscopy revealed the cells that possessed sufficient sensor expression for imaging, and further examination identified adequate numbers of native, polarized cells with active, ruffling leading edges suitable for studies of the effects of modulators on leading edge signaling. The ability to limit the experimental pool to only those native, highly polarized cells with a functional leading edge greatly enhanced the reproducibility of subsequent studies of the effects of modulators on the leading edge signaling pathway.

### The five modulators employed

Previous live cell studies have demonstrated the usefulness of five modulators—two activators and three inhibitors—to perturb leading edge physiology. Activators stimulate the positive feedback loop and trigger leading edge expansion, while inhibitors downregulate the feedback loop and trigger leading edge contraction. **(i)** *Platelet-derived growth factor (PDGF)* is a widely used attractant in live cell studies. PDGF binds to and activates the PDGF receptor (PDGFR), a receptor tyrosine kinase (RTK) that serves as a chemotaxis receptor and directs leukocyte migration to sites of inflammation in animal models and humans [45,46]. Activated PDGFR can directly bind and activate PI3K, thereby triggering increased PIP_3_ production at the leading edge membrane [47,48]. **(ii)** *ATP* is known to activate a global Ca^2+^ signal by binding multiple receptors, including both P2X receptors (cation channels including isoforms that provide direct, rapid, ATP-triggered Ca^2+^ influx) and P2Y receptors (GPCRs that trigger slower downstream Ca^2+^ release from intracellular stores) [49–54]. In addition to direct and indirect stimulation of Ca^2+^ signals, ATP can also serve as a leukocyte attractant [50–52]. **(iii)** *Wortmannin* is a cell permeant PI3K suicide inhibitor known to block PIP_3_ production and the leading edge feedback loop [14,55–57]. **(iv)** *EGTA* is an extracellular Ca^2+^ chelator used to inhibit Ca^2+^ influx through plasma membrane Ca^2+^ channels, and is also known to inactivate the leading edge feedback loop since the Ca^2+^ influx is an essential component [14,58]. **(v)** *Go6976* is a PKC inhibitor known to block the kinase activity of conventional (Ca^2+^-dependent) PKCs [59], including PKCα which has been shown to be highly localized to the leading edge membrane of polarized macrophages [14]. Evidence that such inhibition blocks the leading edge feedback loop has not yet been published, however.

To directly test the specificity of wortmannin and Go6976 as inhibitors of PI3K and PKC, respectively, both inhibitors were tested in quantitative assays of PI3Kα lipid kinase activity and PKCα protein kinase activity, respectively. Each inhibitor effectively blocked the activity of its target enzyme and had no detectable effect on the activity of the other enzyme (see S1 and S2 Figs).

### Effects of modulators on leading edge area

**Fig 2:**
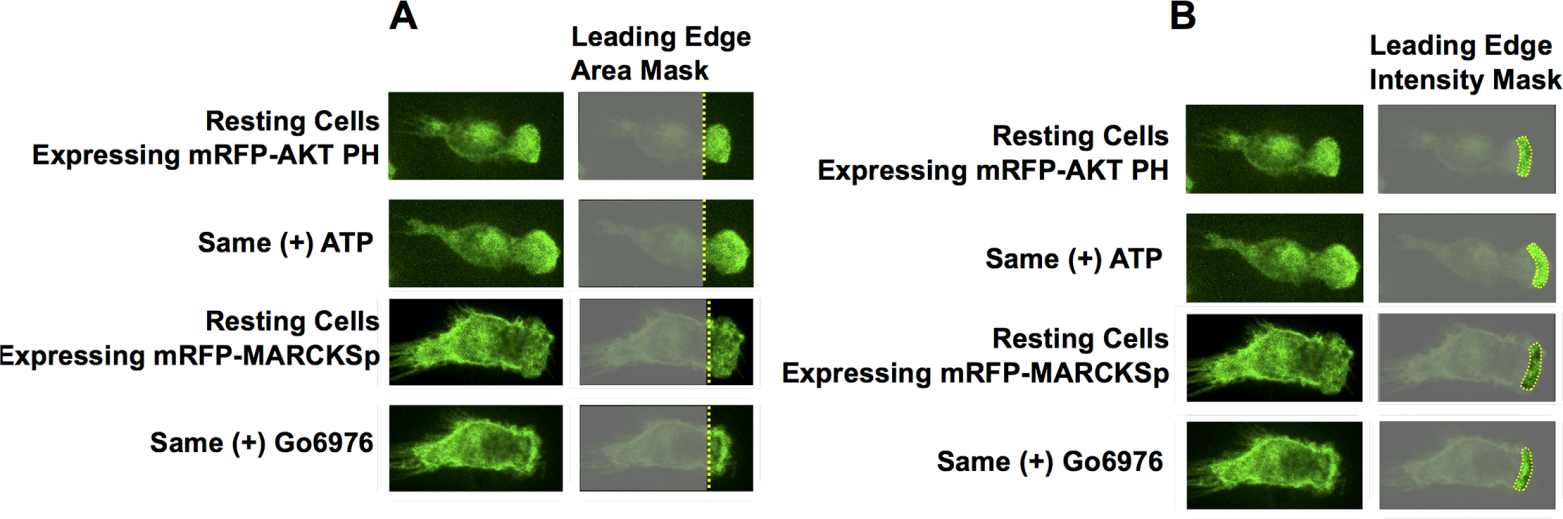
Strategy employed to quantify modulator-triggered changes in the leading edge area, or in key leading edge signaling reactions. Representative pair of RAW 264.7 cells treated with activator (ATP) in the top two rows and inhibitor (Go6976) in the bottom two rows, showing the masks used to quantify leading edge area and activity changes. **(A)** Leading edge *area* changes were determined by first outlining the leading edge region using the freehand selection tool in FIJI, while excluding the bulk of the cell body. The mask baseline (yellow line) was added at the base of the actively ruffling leading edge membrane prior to modulator addition. Changing leading edge area subsequent to addition of modulator, or modulator vehicle, was measured relative to that baseline at time = 0. **(B)** Leading edge *activity* changes were quantified by measuring sensor fluorescence (XFP sensor or CKAR or CellMask) within a defined boundary, depicted by the yellow outline enclosing a portion of leading edge membrane and adjacent cytoplasm. As the timecourse progressed, the mask was manually moved in order to remain proximal to the leading edge boundary. Additional details in Methods.

**Fig 3:**
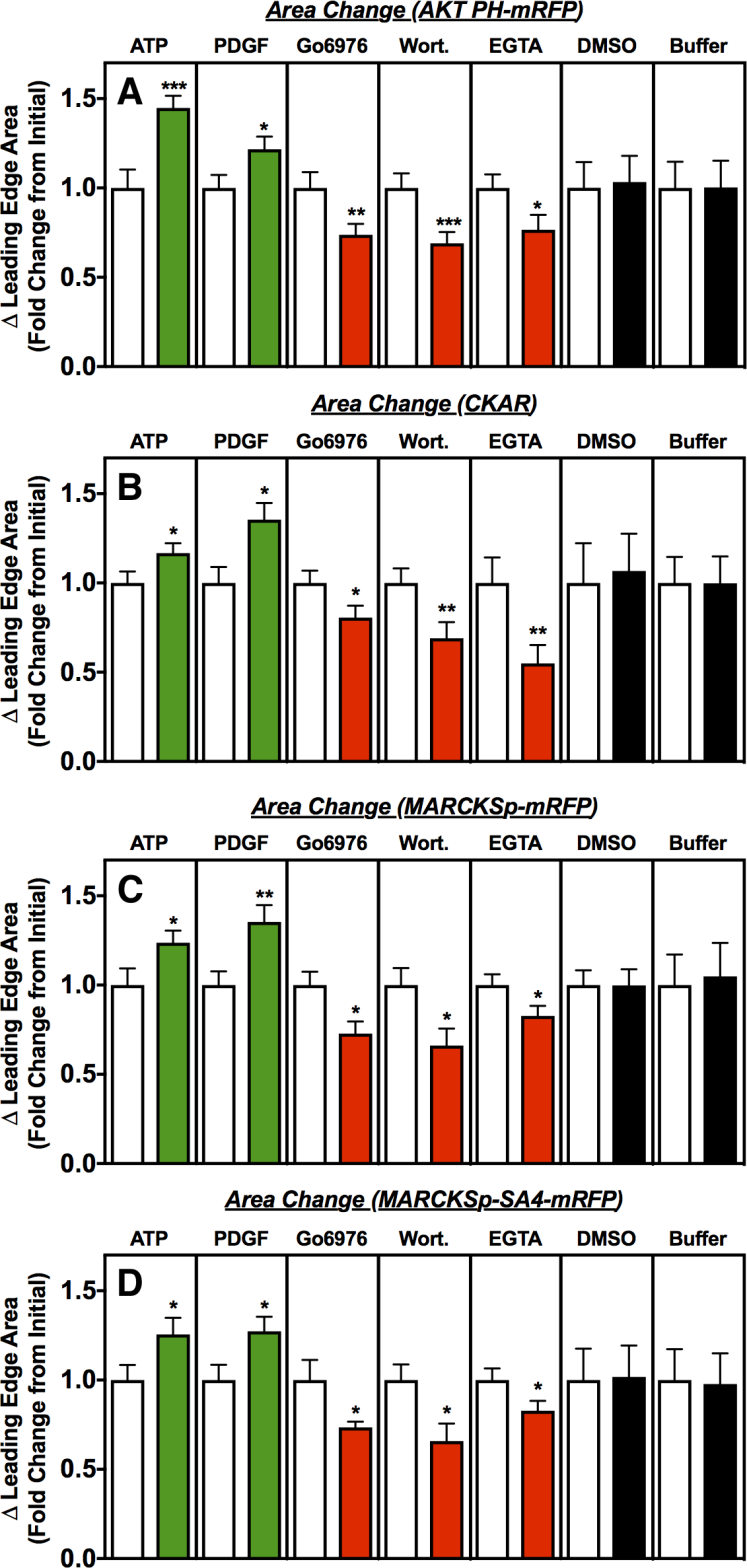
Steady-state changes in leading edge area following modulator addition. Polarized, actively ruffling RAW macrophages expressing the indicated activity sensor were imaged at specific times following addition of the indicated modulators (green = activator, red = inhibitor, black = carrier medium control). Open bars represent the initial leading edge area, normalized to 1.0, immediately after modulator addition. Filled bars represent the fold change as the leading edge area approaches a new steady state size approximately 5 min after modulator addition (t = 4.5 to 5.5 min (see Methods)). As expected for functional leading edge signaling in all sensor backgrounds, activators trigger significant leading edge expansion, inhibitors trigger significant leading edge contraction, and controls have no significant effect. Error bars represent standard errors of the mean for 15–35 cells measured in at least 4 independent experiments. Asterisks indicate significance of each change from the initial area at t = 0 (one, two or three asterisks indicate p < 0.05, p < 0.01, or p < 0.001, respectively). Image analysis described in Fig 2A and Methods.

The effects of each modulator on leading edge area was determined by imaging the functional leading edge regions of robust, highly polarized macrophages exhibiting actively ruffling, leading edge membranes (Fig 2A). As previously observed in the literature [14,51,60–63], Fig 3 shows that the activators PDGF and ATP trigger leading edge expansion, while the inhibitors wortmannin, EGTA and Go6976 yield leading edge contraction. Within error, at 5.5 min after addition, the activators yield the same extent of steady state leading edge expansion, and the inhibitors yield the same extent of contraction. Control additions of the solvent or media (buffer) used to introduce the stock modulator solution have no significant effect. It follows that the macrophages employed in this study possess a functional leading edge signaling circuit, including a regulated positive feedback loop, and that standard activators and inhibitors regulate the feedback loop as expected to drive expansion or contraction of the leading edge, respectively. The observation that the PKC inhibitor Go6976 collapses the leading edge provides new evidence supporting the hypothesis [8,14,20] that conventional PKCs, in particular PKCα, are an essential component of the leading edge feedback loop.

Notably, the same effects of modulators on leading edge area are observed, within error, in cells expressing each of the different fluorescent sensors (Fig 3). These observations indicate that intracellular expression of each fluorescent sensor retains the functional leading edge signaling circuit and its native ability to be regulated by modulators. The same cells and movies employed to analyze modulator effects on leading edge area were next analyzed to determine the effects of each modulator on the leading edge signal of each sensor.

### Effects of modulators on leading edge PI3K activity and PIP_3_ production

**Fig 4:**
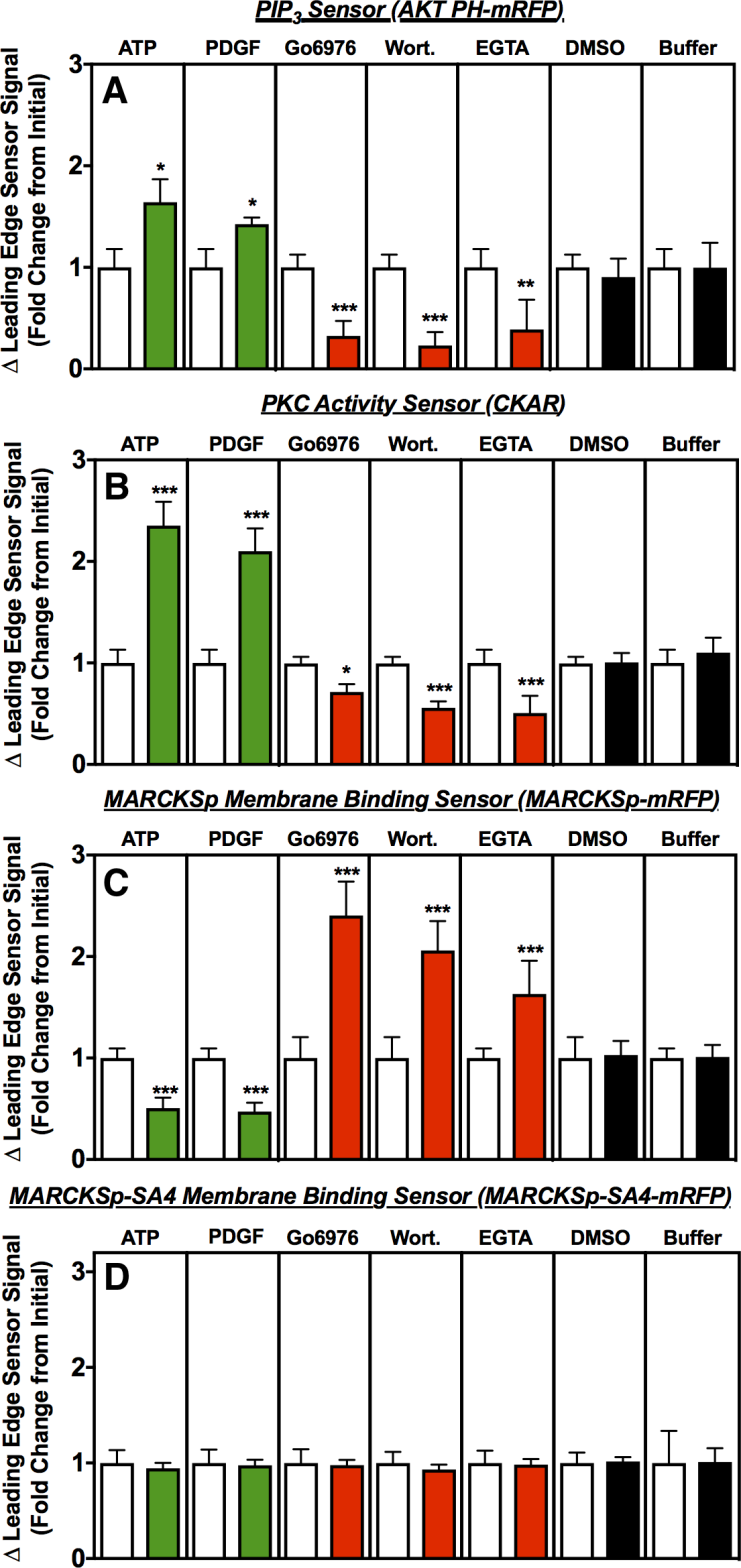
Steady state changes in leading edge activity sensors following modulator addition. The same polarized, actively ruffling RAW macrophages imaged in Fig 3 were also monitored for leading edge signaling activities as detected by the indicated activity sensor at specific times following addition of the indicated modulators (green = activator, red = inhibitor, black = carrier medium control). At each timepoint, the fluorescence signal of the sensor was measured to quantify the leading edge activity it monitors. Open bars represent the initial leading edge activity, normalized to 1.0, immediately after modulator addition. Filled bars represent the fold change as the activity approaches a new steady state level approximately 5 min after modulator addition (t = 4.5 to 5.5 min (see Methods)). The findings indicate that activators significantly increase (and inhibitors significantly decrease) the leading edge PIP_3_ density sensed by AKTPH-mRFP, and the leading edge PKC activity sensed by CKAR. In contrast, the opposite significant changes are observed for MARCKS binding to the leading edge membrane sensed by MARCKSp-mRFP. No significant changes in leading edge membrane binding were observed for the MARCKSp-SA4-mRFP sensor that lacks the Ser residues required for phosphoregulation by PKC. Error bars represent standard errors of the mean for 15–35 cells measured in at least 4 independent experiments. Asterisks indicate significance of each change from t = 0 (one, two or three asterisks indicate p < 0.05, p < 0.01, or p < 0.001, respectively). Image analysis described in Fig 2B and Methods.

The effects of modulators on leading edge activity sensors were quantified in polarized, actively ruffling cells as illustrated in Fig 2B (above). Fig 4A shows the effect of each modulator on leading edge PI3K activity, as measured by the steady state level of product PIP_3_ lipid at the leading edge membrane 5.5 min after modulator addition. As previously observed for leukocyte attractants and Ca^2+^ influx activators [14,64,65] PDGF or ATP strongly enhance recruitment of the PIP_3_ sensor (AKT-PH-mRFP) to the leading edge membrane, indicating that leading edge PIP_3_ production and accumulation is significantly increased. This stimulation of the leading edge PIP_3_ signal is consistent with the known ability of both attractant and Ca^2+^ to activate the leading edge positive feedback loop and thus stimulate PI3Kα lipid kinase activity and PIP_3_ production [14,31,51,66–68]. By contrast, PI3K inhibitor (wortmannin) or Ca^2+^ influx inhibitor (EGTA) trigger decreased recruitment of the PIP_3_ sensor, indicating that leading edge PI3K activity is inhibited by these drugs as previously shown [14]. Not previously published is the effect of PKC inhibitor (Go6976) on the leading edge PIP_3_ signal. This inhibitor significantly reduces the leading edge PIP_3_ signal, providing additional strong evidence supporting the hypothesis [8,14,20] that PKC and PI3K are linked in the leading edge positive feedback loop.

### Effects of modulators on leading edge PKC activity

While previous studies have shown that attractant and Ca^2+^ signals recruit PKC to the leading edge membrane [14], their effect on PKC kinase activity have not been determined. Fig 4B illustrates the effect of each modulator on PKC activity in the vicinity of the leading edge membrane. The attractant (PDGF) and Ca^2+^ influx (ATP) activators yield increased PKC-specific phosphorylation of the CKAR FRET sensor at the leading edge, as detected by a characteristic change in intramolecular FRET upon sensor phosphorylation. By contrast, the Ca^2+^ influx inhibitor (EGTA) and PKC inhibitor (Go6976) trigger decreased PKC activity and CKAR phosphorylation as expected since these inhibitors block the Ca^2+^ and ATP loading, respectively, needed to activate PKC. Similarly, the PI3K inhibitor (wortmannin) inhibits the leading edge PKC activity, providing strong new support for the linkage between PKC and PI3K in the positive feedback loop [8,14,20].

### Effects of modulators on MARCKS recruitment to the leading edge

The binding of MARCKS to PIP_2_ at the leading edge of macrophages has not previously been investigated. Fig 4C presents the effect of each modulator on the recruitment of the soluble, fluorescent MARCKS peptide (MARCKSp-mRFP) to the leading edge membrane. Ca^2+^-activated PKC is known to displace MARCKS from its tightly bound PIP_2_ by phosphorylating its PIP_2_ binding region [20,23,34,38,40,41]. The attractant (PDGF) and Ca^2+^ influx (ATP) activators that enhance PKC activity, as shown above (Fig 4B), are observed to yield displacement of MARCKSp-mRFP from the leading edge membrane, while the PKC inhibitor (Go6976), Ca^2+^ influx inhibitor (EGTA) and PI3K inhibitor (wortmannin) each yield increased recruitment of MARCKSp-mRFP to the leading edge membrane (Fig 4C).

In contrast, modulators have no effects on the membrane localization of a control sensor (MARCKSp-SA4-mRFP) in which all four Ser residues serving as specific PKC phosphorylation sites are mutated to Ala (Fig 4D), confirming that membrane displacement requires PKC-catalyzed MARCKSp phosphorylation. Notably, Ca^2+^-CaM can also bind MARCKS and displace it from the membrane [26,69,70]. The Ser-Ala mutations of MARCKSp-SA4-mRFP do not alter CaM recognition residues and thus are not thought to decrease CaM binding [25]. **(**Instead, these mutations increase local hydrophobicity and may modestly increase the affinity of CaM binding [25]). Thus, the observation that activators and inhibitors have strong effects on MARCKSp-mRFP membrane binding, but not on that of MARCKSp-SA4-mRFP, supports a model in which modulator-regulated of PKC phosphorylation (not Ca^2+^-CaM) controls the PIP_2_ and membrane binding of MARCKSp-mRFP at the leading edge of stably polarized, immobilized macrophages.

### Effects of modulators on timecourses of leading edge area and activities

**Fig 5:**
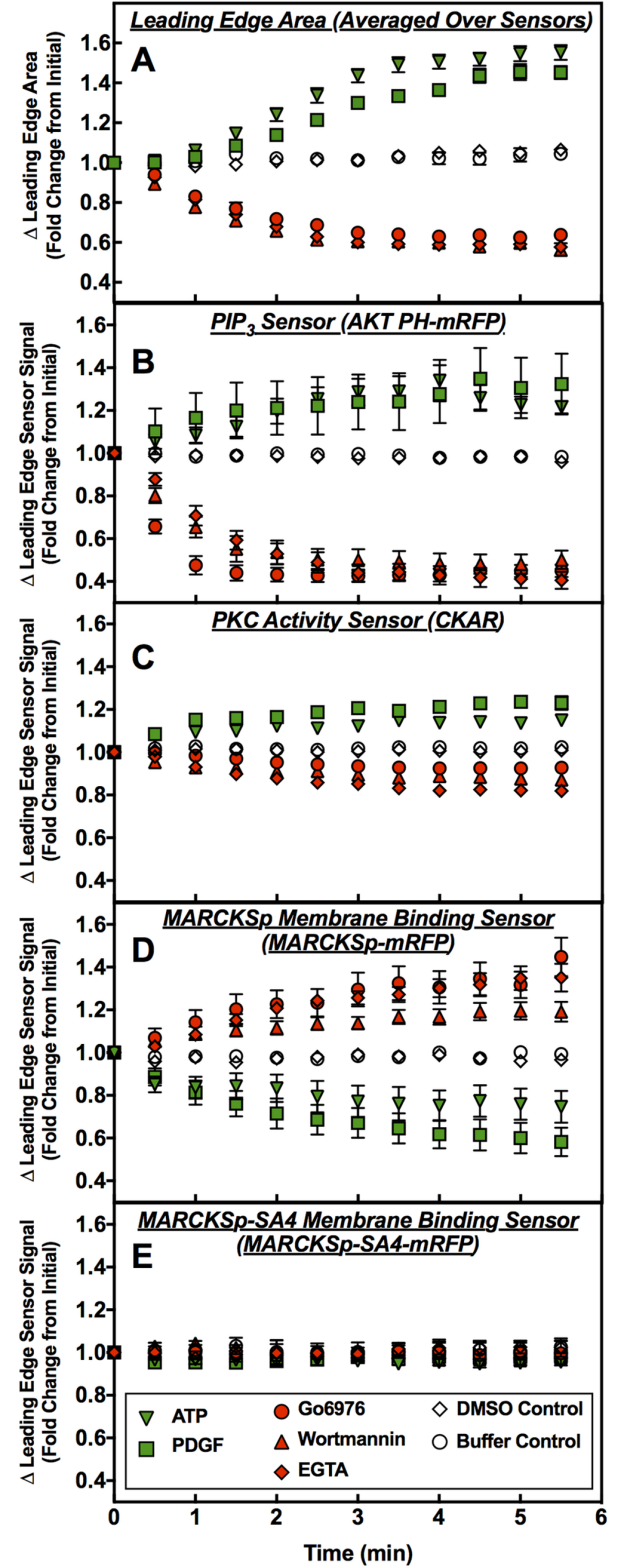
**Timecourses of leading edge area changes (A) and leading edge activity changes (B-E) following modulator addition.** Timecourses were measured for same polarized, actively ruffling RAW macrophages imaged in Figs 2 and 3 (see those figure legends for additional details) following addition of the indicated modulators (green = activator, red = inhibitor, open = carrier medium control). (A) The area change data indicate that the leading edge area expansion triggered by activators is slower, and appears to exhibit biphasic kinetics with a lag phase, compared to the more monophasic contraction triggered by inhibitors. (B-D) The most rapid leading edge activity changes are observed for the inhibitor-triggered decreased in PIP_3_ density sensed by AKTPH-mRFP, while the slowest change is observed for the attractant PDGF-triggered dissociation of MARCKSp-mRFP from the leading edge membrane. Error bars represent standard errors of the mean for 15–35 cells measured in at least 4 independent experiments.

Fig 5 shows the effects of modulators on the timecourses of leading edge area changes and activity changes, respectively. Fitting of the timecourses with simple kinetic models was not attempted, since the reactions and kinetics underlying leading edge area and activity changes are undoubtedly complex and the data do not have sufficient resolution to resolve alternative models.

Fig 5A illustrates the timecourses of area changes following addition of each modulator. Since the timecourse observed for a given modulator was found to be largely independent of the sensor background, each timecourse is averaged over different backgrounds. Notably, after addition of either activator (PDGF or ATP), the resulting leading edge expansion exhibits a clearly biphasic timecourse with initial slow expansion followed by faster expansion. In contrast, after addition of inhibitor (wortmannin, EGTA or Go6976), the leading edge contraction exhibits a simpler, more monophasic timecourse approximately 2-fold faster than that observed for activator-triggered expansion.

Fig 5B–5E show the timecourses of sensor fluorescence changes following addition of each modulator. Here the separate timecourses are shown for each sensor since different sensors display contrasting kinetics. To a first approximation, for each sensor the two activators (or the three inhibitors) exhibit similar kinetics even when their final amplitudes differ slightly. The timecourses are fastest for inhibitor-triggered losses of leading edge PIP_3_ as sensed by AKT-PH-mRFP, which approach a new, lower steady state level within ~2 min. The slowest changes are observed for MARCKSp-mRFP membrane association, especially for attractant-triggered dissociation from membrane that may take as long as 5+ min to approach a new steady state.

## Discussion

The present findings fully support the Ca^2+^-PKCα-MARCKS-PIP_2_-PI3K-PIP_3_ signaling module hypothesis [8,14,20]. Activators are observed to increase leading edge PKCα activity, displace MARCKS from the leading edge, increase leading edge PI3K activity and PIP_3_ production, and stimulate expansion of the leading edge region. These results are predicted by the model, in which activators stimulate the leading edge positive feedback loop, thereby (i) increasing the local Ca^2+^ signal and PKC activity, which in turn (ii) increases MARCKS phosphorylation and displaces MARCKS from its PIP_2_ binding sites on the membrane, thereby releasing sequestered PIP_2_ that (iii) increases net PI3K activity and production of PIP_3_. Together, (iv) the increased PKC activity, PIP_2_ availability, and PIP_3_ production drive expansion of the leading edge region via complex downstream reactions that expand the actin mesh and membrane surface area. At the other extreme, inhibitors of the Ca^2+^-PKCα-MARCKS-PIP_2_-PI3K-PIP_3_ signaling module are observed to decrease PKC activity, increase MARCKS binding to the membrane, and decrease PIP3 production, thereby yielding contraction of the leading edge region as the model predicts [8,14,20].

The findings include new evidence supporting the linkage of PKC and PI3K signaling in the leading edge positive feedback loop [8,14,20], since activators of PI3K signaling (PDGF, ATP) also activate PKC and phosphorylation of PKC substrates (CKAR and MARCKSp-mRFP). Moreover, a specific PI3K inhibitor (wortmannin) inhibits not only PIP3 production, but also inhibits PKC activity as detected by decreased phosphorylation of its substrates. Similarly, a specific PKC inhibitor (Go6976) not only inhibits PKC activity, but also blocks leading edge PI3K activity. These new observations fit well with previous findings that Ca^2+^ influx and PKCα are strongly localized to the leukocyte leading edge, where they are both essential components of the leading edge positive feedback loop [14, 15].

The new results further suggest that at the leading edge of polarized, actively ruffling macrophages, Ca^2+^-PKC kinase activity rather than Ca^2+^-CaM binding dominates MARCKS regulation of free PIP_2_ density. *In vitro* studies have shown that both Ca^2+^-PKC-catalyzed MARCKS phosphorylation and Ca^2+^-CaM binding to MARCKS can displace MARCKS from PIP_2_, thereby increasing the density of free PIP_2_ and net PI3K activity [20,26]. The four Ser to Ala mutations of the MARCKSp-SA4-mRFP construct eliminate all four PKC phosphorylation sites, and thus should block Ca^2+^-PKC regulation, but do not modify the CaM recognition sequence and thus should not disrupt Ca^2+^-CaM regulation [25]. The MARCKSp-SA4-mRFP construct is observed to be targeted to the leading edge membrane, indicating it retains PIP_2_ binding as expected [21,41,71], but all regulation of membrane binding-dissociation is lost, providing strong evidence that Ca^2+^-PKC kinase activity, rather than Ca^2+^-CaM binding, dominate MARCKS regulation under these cellular conditions. The *in vitro* studies have demonstrated that Ca^2+^-PKC phosphorylation of the MARCKS population is slow, on the timescale of approximately 2–5 min at physiological concentrations, while Ca^2+^-CaM binds MARCKS in seconds [20,26]. It therefore remains possible that fast Ca^2+^-CaM regulation of MARCKS is important in gradient detection and chemotactic migration on attractant trails, while the slow Ca^2+^-PKC regulation of MARCKS observed herein dominates in the steady state leading edge pathway operating in the absence of an attractant gradient (the present studies apply no external spatial gradients to the cells). Further studies are needed in cells migrating up attractant gradients to ascertain whether Ca^2+^-CaM plays a role in the rapidly responding compass that guides chemotactic behavior.

Certain elements of the observed Ca^2+^-PKC-MARCKS-PIP_2_-PI3K-PIP_3_ signaling module may well be universally important in eukaryotic cell chemotaxis, while other features may be unique to leukocytes due to their advanced, highly specialized gradient sensing and chemotaxis. In mammalian systems, the chemotaxis pathways of leukocytes (ameboid cells) and fibroblasts (mesenchymal cells) are best studied. It now appears likely these ameboid and mesenchymal pathways likely share a PLC-DAG-Ca^2+^-PKC-MARCKS-PIP_2_ signaling module that includes phospholipase C (PLC) to generate the diacylglycerol (DAG) needed to activate the kinase activity of leading edge conventional Ca^2+^-PKCs. In fibroblasts DAG, as well as other elements of the PLC-DAG-Ca^2+^-PKC-MARCKS-PIP_2_ signaling module, have been observed to be localized at the leading edge [16–19]. DAG has not yet been imaged in leukocytes, but is also predicted to exhibit leading edge localization. In both ameboid and mesenchymal cells, the proposed PLC-DAG-Ca^2+^-PKC-MARCKS-PIP_2_ signaling module likely generates positive feedback since the output PIP_2_ is a substrate for PLC in DAG production [72], and is also a known enhancer of conventional PKC membrane binding affinity, bound state lifetime, and catalytic activity [73–79]. In fibroblasts, PI3K-generated PIP_3_ signals are not essential for chemotaxis [16–19], in contrast to the essential nature of PIP_3_ signals in leukocyte chemotaxis [6,14,80,81]. Thus, in leukocytes the leading edge signaling module is proposed to include the essential components PLC-DAG-Ca^2+^-PKC-MARCKS-PIP_2_-PI3K-PIP_3_, while in mesenchymal cells the simpler PLC-DAG-Ca^2+^-PKC-MARCKS-PIP_2_ module appears to suffice. Much remains to be learned about key regulatory mechanisms within these leading edge modules, as well as the upstream receptor-triggered reactions that activate the modules, and the downstream reactions controlled by the modules to regulate actin and membrane remodeling in directed cell migration.

More broadly, PIP_3_ signaling regulates other key cell pathways, including and excessive PIP_3_ production has long been linked to many human cancers (reviewed in [82]). Recently, a link between carcinogenesis and excessive phosphorylated MARCKS has also emerged (reviewed in [83]). The present findings reveal a simple molecular mechanism hypothesized to underlie the ability of excessive phospho-MARCKS to trigger cancer, in which increased levels of phospho-MARCKS trigger higher free PIP_2_ levels that recruit additional active PI3K to the membrane, thereby increasing net PIP_3_ production.

## Materials and methods

### Materials

RAW 264.7 murine macrophage cells were obtained from the American Type Culture Collection (Manassas, VA). These cells (lot 70000171) were analyzed by ATCC and confirmed as RAW 264.7 based on morphology, growth properties and test for contamination. Cells were typically passaged 6–10 times, never more than 12 times. Only cells retaining characteristic, strong leukocyte polarization with a ruffling leading edge were utilized in the studies described herein. Wortmannin, ATP, PDGF-ββ, EGTA and HEPES were from Sigma (St. Louis, MO). Go6976 was from Tocris Bioscience (Minneapolis, MN). Neon Transfection System, anhydrous DMSO and CellMask Green and Deep Red plasma membrane stains were from Thermo Fischer Scientific (Waltham, MA). Cell culture media components included FluoroBrite DMEM media (Thermo Fischer Scientific (Waltham, MA)), fetal bovine serum (FBS) from Sigma (St. Louis, MO), Pen Strep (penicillin streptomycin) from Gibco (Gaithersburg, MD) and GlutaminePlus (L-alanyl-L-glutamine) form Atlanta Biologicals (Flowery Branch, GA). Matriplate plates with 96 culture wells and 0.17mm glass bottom were from Matrical, Inc (Spokane, WA). C kinase activity reporter (CKAR) mammalian expression plasmid was a kind gift from the lab of Alexandra C. Newton [37]. AKT-PH domain was subcloned from IMAGE clone 4562823 (residues 1–120) into pmRFP1(C3) (Invitrogen) by John H. Evans to generate mammalian expression plasmid AKT-PH-mRFP [14]. Mammalian expression plasmids for MARCKSp-mRFP and MARCKSp-SA4-mRFP constructs were gifts from the lab of Barbara Baird [42].

### Cell culture, transient transfection, and preparation for imaging

RAW264.7 cells were cultured and passaged at 37°C, 5% CO_2_ to ~80% confluency in DMEM media supplemented with 10% heat-inactivated FBS, 20mM HEPES, 2 mM GlutaminePlus and 100 U/mL penicillin, 100 *μ*g/mL streptomycin. Cells were washed in room-temperature Dulbecco's PBS (D-PBS), scraped in a volume of room temperature 10 mL D-PBS and counted using a hemocytometer. After counting, cells were pelleted and resuspended to a concentration of 5x10^6^ cells/mL in prewarmed (37°C) Neon Buffer T and mixed with 30μg of the relevant reporter-XFP plasmid (plasmid addition diluted the cell suspension ≤10%). The cell and plasmid DNA suspension were aspirated using 100 μL Neon tips and electroporated using the Neon system set for a single pulse at 1680 volts for 20 ms. Electroporation mixtures were immediately mixed into 15 mL of prewarmed (37°C) antibiotic-free DMEM supplemented with FBS, 20mM HEPES and L-glutamine dipeptide. 0.5 mL of the resulting cell suspension was added to 0.6 mL capacity Matriplate wells and incubated for 4 hours at 37°C, 5% CO_2_, whereupon the media was aspirated and replaced with prewarmed (37°C) DMEM supplemented with FBS, HEPES, L-glutamine and antibiotics as described above. Transfected cells were incubated at 37°C, 5% CO_2_ for at least 24, and up to 48 hours after transfection.

At least three hours before imaging experiments, cells were rinsed twice with prewarmed (37°C) FluoroBrite DMEM supplemented with 10% heat-inactivated FBS, 20mM HEPES, 0.292 mg/mL L-glutamine dipeptide and 100 U/mL penicillin, 100 *μ*g/mL streptomycin. Cells were then allowed to acclimate at 37°C, 5% CO_2_ for 3–4 hours. Where indicated, prior to imaging, adherent cell plasma membranes were stained for 10 minutes at 37°C with a 1:1000 dilution of CellMask dye, diluted into Fluorobrite DMEM with supplements described above, followed by three aspiration-rinse cycles using prewarmed (37°C) FluoroBrite DMEM with supplements.

### Imaging and microscopy

Live-cell microscopy experiments were carried out on a Nikon TiE Confocal microscope equipped with a Nipkow Yokogawa CSU-X1 spinning disc unit, a 60x 1.4 N.A, oil immersion objective, seven laser lines and an Andor iXon Ultra 888 EMCCD camera. The excitation laser lines used for imaging were: 488 nm (CFP), 514 nm (YFP), 561 nm (CellMask Green plasma membrane stain), 594 nm (RFP), 640 nm (CellMask Deep Red plasma membrane stain). The quad emission filters employed were: 447/38 nm (CFP), 520/22 nm (YFP and cellmask green), 598/40 nm (RFP), 705/68 nm (cellmask deep red). The microscope was fully enclosed in a humidified Oko Labs environmental chamber at 37°C, 5% CO_2_. Cells selected for imaging and further experimentation were both (i) visibly expressing the transfected fluorescent fusion protein, and (ii) clearly polarized and displaying an actively ruffling leading edge. After an initial image was collected, a selected drug, or control drug vehicle (FluoroBrite DMEM or DMSO), was mixed into the media at zero time and data collection was initiated at t = 30 sec. At 30 second increments, a 0.5 μm Z-stack was collected using Nikon Elements, typically resulting in a Z-stack of 11 images. Exposure time for acquisitions were typically set as follows: YFP 300 ms, RFP 300 ms, GFP 300 ms and CFP 500 ms. Final drug concentrations were: PDGFββ 2.1 μM (equivalent to 50 ng/ml, added in DMEM supplemented as indicated above); ATP 25 μM (added in DMEM supplemented as indicated above); EGTA 3 mM (added in DMEM supplemented as indicated above); wortmannin 500 nM (added in DMSO); Go6976 1 μM (added in DMSO).

### Image analysis

Images were analyzed with Fiji, the open-source processing program distribution of ImageJ [84]. A Z-projection transformation was applied to all Z-stacks to convert the three-dimensional image dataset into a single two-dimensional image. The sum-type projection algorithm (versus average- or maximum-type projections) offered the clearest two-dimensional image conversion with the least ambiguity (clear resolution of cell boundaries and internal compartmentalization). Determination of cellular leading edge area change was determined according to the schematic presented in Fig 2. The freehand selection tool in FIJI was used to outline the leading edge, while excluding the bulk of the cell body. This mask was added as a region of interest to the ROI manager and set as the back edge of the resting leading edge boundary. Analysis of XFP or CellMask plasma membrane stain fluorescence distribution in RAW 264.7 cells was performed by integrating the background- and bleach-corrected fluorescence from the leading-edge membrane region (indicated in Fig 2). First, the freehand selection tool was used to mark a section of leading edge membrane and adjacent cytoplasm (yellow outline in Fig 2B). On a cell-specific basis, care was taken with each mask to ensure that cell and/or leading edge movement did not displace the mask from the leading edge. In some cases, the mask was manually moved as needed to track a dynamic leading edge. After a background subtraction (bleach correction), integrated fluorescence change with respect to time was normalized to the fluorescence values of the leading-edge region at time zero. Final, steady state area and activity values achieved after modulator addition were determined by averaging the data for timepoints at 4.5, 5.0 and 5.5 minutes (there was little difference between those timepoints). All indicated errors for initial and steady state values, and for timepoints, represent standard errors of the mean for 10–30 cells (or 7–17 cells for controls) measured in at least 4 independent experiments.

For the simple fluorescence sensors employed (AKT-PH-mRFP, MARCKSp-mRFP, MARCKSp-SA4-mRFP) the above procedure yielded the fractional change in the leading edge signal, while for the FRET sensor CKAR additional data analysis was needed. The soluble, cytoplasmic FRET sensor CKAR was imaged near the leading edge membrane (see above) since the plasma membrane-targeted PMCKAR did not yield adequate signal to detect PKC activity changes. The CKAR FRET signal *decreases* as PKC kinase activity *increases* the steady state phosphorylation level of the CKAR population. To generate a parameter proportional to PKC kinase activity, a standard procedure was employed [37]. Image stacks generated with donor CFP excitation (488 nm) were separated by Fiji into their CFP donor emission and YFP acceptor emission channels, which were then used to calculate the ratio (CFP donor emission) / (YFP acceptor emission). The resulting ratio increases when increasing PKC kinase activity shifts the CKAR population towards higher phosphorylation levels [37]. Figures present the fractional change in this PKC activity parameter.

## Supporting information

S1 Fig Evaluation of inhibitor specificity using single molecule TIRFM assay for PI3K lipid kinase activity. To compare and quantify the inhibitory effects of wortmannin and Go6976 on PI3K lipid kinase activity, our previously described single molecule TIRFM assay [20, 26, 85] was used to monitor PI3K activity on supported lipid bilayers by counting each PIP_3_ product molecule generated via the binding of a high affinity fluorescent PIP_3_ sensor (GRP PH domain labeled with AF 555). (**A**) Representative single particle tracks for GRP1 PH-PIP_3_ complexes. Such tracks were evaluated and identified by a stringent set of criteria based on size, brightness and diffusion speed to count the number of product PIP3 molecules generated by PI3K as a function of time. **(B)** Timecourse of PI3K-catalyzed PIP_3_ production showing the linear accumulation with time of single particle tracks identified as fluorescent GRP PH-PIP_3_ complexes. The timecourse of PIP_3_ production is slowed dramatically by the PI3K inhibitor wortmannin, but not by the PKC inhibitor Go6976. **(C)** The rate of each reaction in (B) determined from the slope of its timecourses. The rates confirm that the PKC-specific inhibitor Go6976 has no significant effect on PI3K kinase activity while the PI3K-specific inhibitor wortmannin efficiently suppresses PIP_3_ production by the lipid kinase. In all cases, error bars are standard errors of the mean (n ≥ 9), and measurements were 21.5 ± 0.5°C in 100 mM KCl, 20 mM HEPES pH 6.9 (optimal pH for PI3K activity), 15 mM NaCl, 5 mM glutathione, 2.0 mM EGTA, 1.9 mM Ca^2+^, 1.9 mM Mg^2+^, 1.0 mM ATP, 100 μg ml^-1^, and 0.05% CHAPS. Under these conditions, the EGTA-ATP-Ca^2+^ buffering system yields 10 μM free Ca^2+^ and 0.5 mM free Mg^2+^.
(TIFF)

S2 Fig Evaluation of inhibitor specificity using bulk assay for PKC kinase activity. To determine the effect of wortmannin and Go6976 on the activity of PKCα, a modified PepTag assay was employed to quantify bulk PKCα activity (Promega, Madison, WI [20, 79]). PKC phosphorylation of a synthetic fluorescent peptide substrate alters the net charge from +1 to -1, allowing for the separation of the phosphorylated and nonphosphorylated versions by electrophoresis on an agarose gel. (**A**) Raw data of separated phosphorylated and nonphosphorylated PKC substrate after a 30 minute incubation at 30°C. (**B**) Optical density of the phosphorylated (lower) bands are quantitated using the ImageJ [86] gel analyzer plugin and allows for comparison of normalized PKC activity in the modified PepTag assay in the presence of PI3K and PKC inhibitors, wortmannin and Go6976, respectively. (**C**) To ensure that the PKC activity remained in the linear, initial rate phase of the reaction, several reaction times between 1–60 min were tested. The reaction was linear for at least 30 min, in accordance with the manufacturer protocol. The rates confirm that the PKC-specific inhibitor Go6976 blocked PKC kinase activity while the PI3K-specific inhibitor wortmannin had little or no effect on PKC. Kinase assays were performed at 30°C per manufacturer protocol, except the PKC lipid activator (phosphatidylserine) was replaced with 200 μg/ml sonicated unilamellar vesicles (SUV) comprised of PC:PS:PIP_2_:DAG (lipids from Avanti Polar Lipids (Alabaster, AL)) at lipid mole percentages of 73:23:2:2, respectively, closely matching the lipid composition employed in the single molecule studies of S1 Fig. Additionally, the manufacturer assay buffer was replaced with a PKC kinase assay buffer (10 mM MgCl_2_, 26 μM CaCl_2_, 20 μM EGTA, 1 mM EGTA, 1 mM DTT, 1 mM ATP and 20 mM HEPES pH 7.4).
(TIFF)
