# Supplementary figures and images for: A PKC-MARCKS-PI3K regulatory module links Ca2+ and PIP3 signals at the leading edge of polarized macrophages
Source: PLoS One. 2018 May 1;13(5):e0196678. doi: 10.1371/journal.pone.0196678 (PMC5929533; doi:10.1371/journal.pone.0196678)

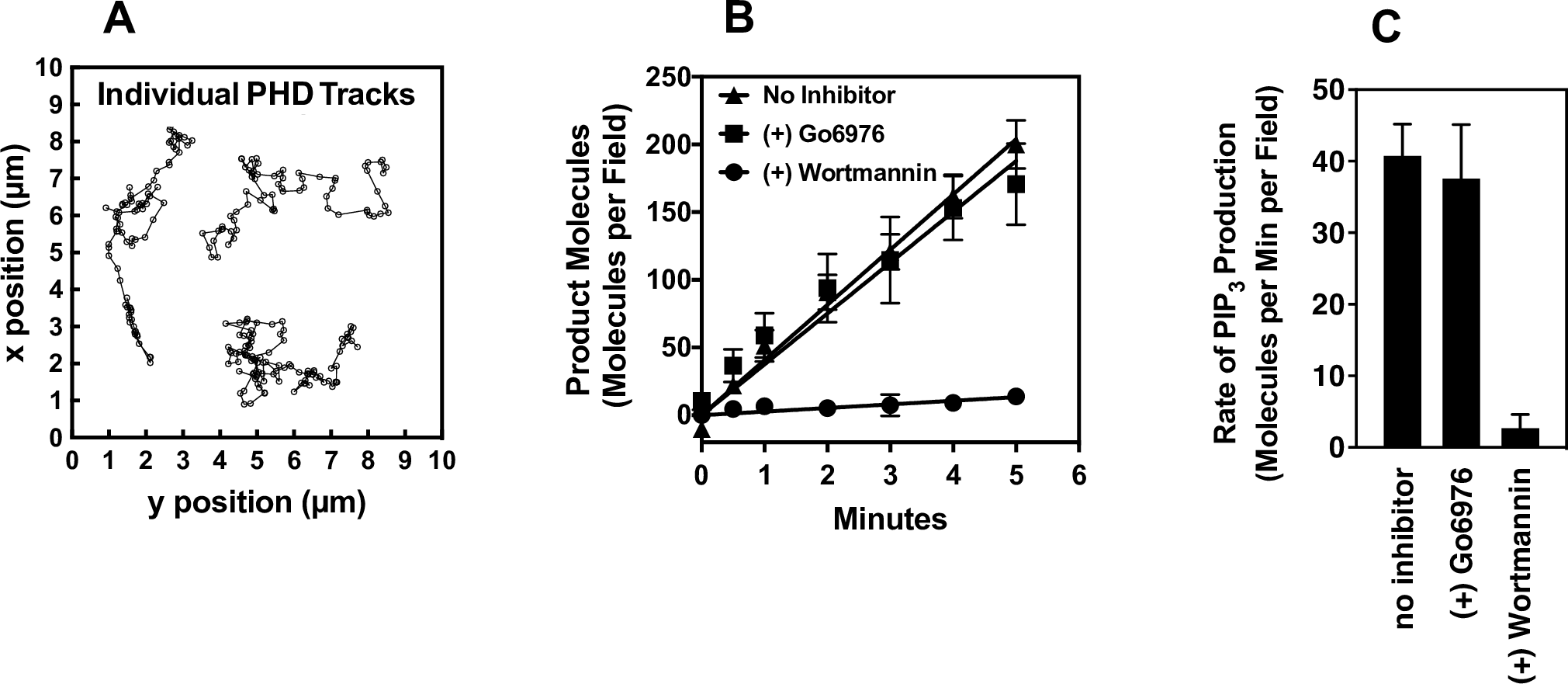

Supplement: S1 Fig — To compare and quantify the inhibitory effects of wortmannin and Go6976 on PI3K lipid kinase activity, our previously described single molecule TIRFM assay [20, 26, 85] was used to monitor PI3K activity on supported lipid bilayers by counting each PIP3 product molecule generated via the binding of a high affinity fluorescent PIP3 sensor (GRP PH domain labeled with AF 555). (A) Representative single particle tracks for GRP1 PH-PIP3 complexes. Such tracks were evaluated and identified by a stringent set of criteria based on size, brightness and diffusion speed to count the number of product PIP3 molecules generated by PI3K as a function of time. (B) Timecourse of PI3K-catalyzed PIP3 production showing the linear accumulation with time of single particle tracks identified as fluorescent GRP PH-PIP3 complexes. The timecourse of PIP3 production is slowed dramatically by the PI3K inhibitor wortmannin, but not by the PKC inhibitor Go6976. (C) The rate of each reaction in (B) determined from the slope of its timecourses. The rates confirm that the PKC-specific inhibitor Go6976 has no significant effect on PI3K kinase activity while the PI3K-specific inhibitor wortmannin efficiently suppresses PIP3 production by the lipid kinase. In all cases, error bars are standard errors of the mean (n ≥ 9), and measurements were 21.5 ± 0.5°C in 100 mM KCl, 20 mM HEPES pH 6.9 (optimal pH for PI3K activity), 15 mM NaCl, 5 mM glutathione, 2.0 mM EGTA, 1.9 mM Ca2+, 1.9 mM Mg2+, 1.0 mM ATP, 100 μg ml-1, and 0.05% CHAPS. Under these conditions, the EGTA-ATP-Ca2+ buffering system yields 10 μM free Ca2+ and 0.5 mM free Mg2+. (TIFF) [file pone.0196678.s001.tiff]

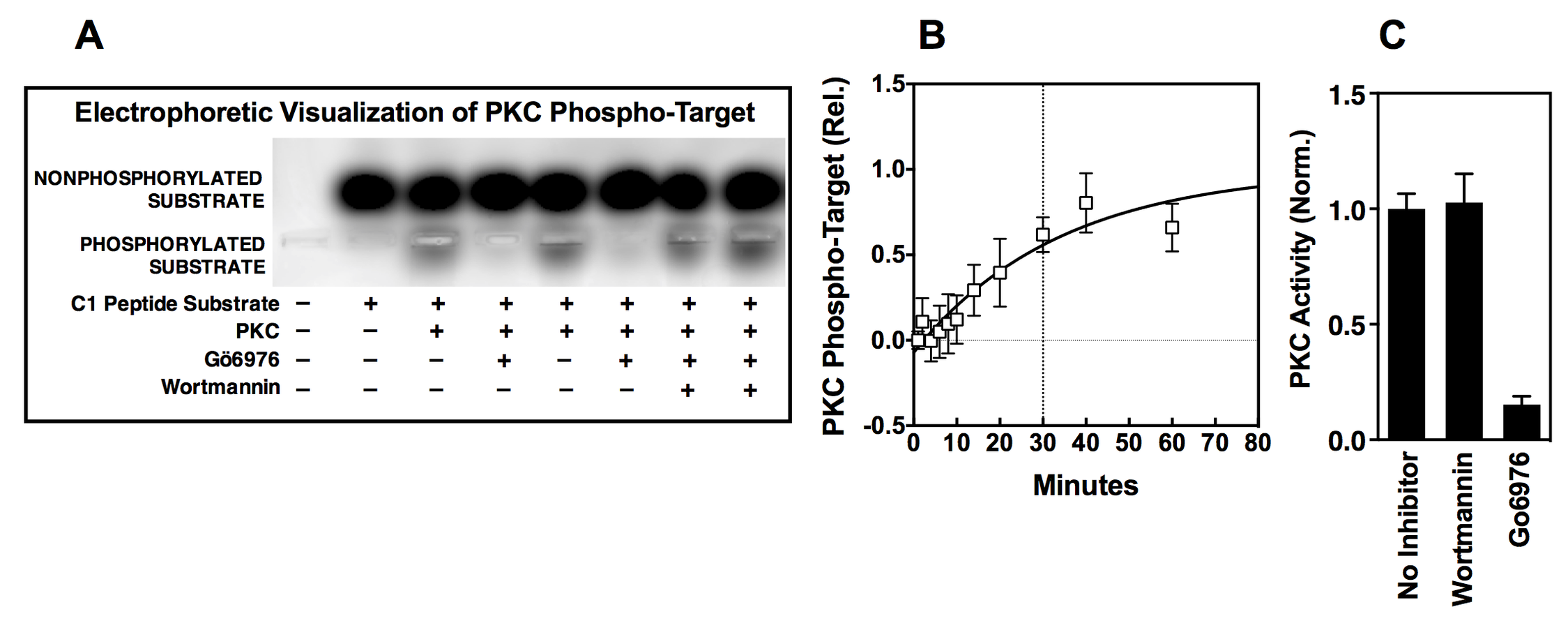

Supplement: S2 Fig — To determine the effect of wortmannin and Go6976 on the activity of PKCα, a modified PepTag assay was employed to quantify bulk PKCα activity (Promega, Madison, WI [20, 79]). PKC phosphorylation of a synthetic fluorescent peptide substrate alters the net charge from +1 to -1, allowing for the separation of the phosphorylated and nonphosphorylated versions by electrophoresis on an agarose gel. (A) Raw data of separated phosphorylated and nonphosphorylated PKC substrate after a 30 minute incubation at 30°C. (B) Optical density of the phosphorylated (lower) bands are quantitated using the ImageJ [86] gel analyzer plugin and allows for comparison of normalized PKC activity in the modified PepTag assay in the presence of PI3K and PKC inhibitors, wortmannin and Go6976, respectively. (C) To ensure that the PKC activity remained in the linear, initial rate phase of the reaction, several reaction times between 1–60 min were tested. The reaction was linear for at least 30 min, in accordance with the manufacturer protocol. The rates confirm that the PKC-specific inhibitor Go6976 blocked PKC kinase activity while the PI3K-specific inhibitor wortmannin had little or no effect on PKC. Kinase assays were performed at 30°C per manufacturer protocol, except the PKC lipid activator (phosphatidylserine) was replaced with 200 μg/ml sonicated unilamellar vesicles (SUV) comprised of PC:PS:PIP2:DAG (lipids from Avanti Polar Lipids (Alabaster, AL)) at lipid mole percentages of 73:23:2:2, respectively, closely matching the lipid composition employed in the single molecule studies of S1 Fig. Additionally, the manufacturer assay buffer was replaced with a PKC kinase assay buffer (10 mM MgCl2, 26 μM CaCl2, 20 μM EGTA, 1 mM EGTA, 1 mM DTT, 1 mM ATP and 20 mM HEPES pH 7.4). (TIFF) [file pone.0196678.s002.tiff]
